# Current Status of ^68^Ga-Pentixafor in Solid Tumours

**DOI:** 10.3390/diagnostics12092135

**Published:** 2022-09-02

**Authors:** Bawinile Hadebe, Machaba Michael Sathekge, Colleen Aldous, Mariza Vorster

**Affiliations:** 1 Department of Nuclear Medicine, College of Health Sciences, University of KwaZulu Natal, Private Bag X54001, Durban 4001, South Africa; 2 Inkosi Albert Luthuli Central Hospital, Durban 4058, South Africa; 3 Department of Nuclear Medicine, Faculty of Health Sciences, University of Pretoria, Pretoria 0002, South Africa; 4 Department of Genetics, College of Health Sciences, University of KwaZulu Natal, Durban 4058, South Africa

**Keywords:** CXCR4, Pentixafor, PET

## Abstract

Chemokine receptor CXCR4 is overexpressed in neoplasms and its expression is related to tumour invasion, metastasis and aggressiveness. ^68^Ga-Pentixafor is used to non-invasively image the expression of CXCR4 in tumours and has been widely used in haematological malignancies. Recent evidence shows that therapies targeting CXCR4 can increase the chemosensitivity of the tumour as well as inhibit tumour metastasis and aggressiveness. ^68^Ga-Pentixafor has shown promise as an elegant radiotracer to aid in the selection of patients whose tumours demonstrate CXCR4 overexpression and who therefore may benefit from novel therapies targeting CXCR4. In addition, its therapeutic partners ^177^Lu- and ^90^Y-Pentixather have been investigated in the treatment of patients with advanced haematological malignancies, and initial studies have shown a good treatment response in metabolically active lesions. ^68^Ga-Pentixafor in solid tumours complements ^18^F-FDG by providing prognostic information and selecting patients who may benefit from therapies targeting CXCR4. This review summarises the available literature on the potential applications of ^68^Ga-Pentixafor in solid tumours.

## 1. Introduction

Cancer is a worldwide public health problem due to its rising prevalence with serious morbidity and high mortality despite advances in surgery and chemotherapy agents. Unfortunately, existing therapies (particularly chemoradiation) are often not successful due to the development of resistance, and oncology patients may have very different outcomes even when receiving the same therapies [1]. Therefore, the one size fits all strategy needs to be replaced with personalised therapies that target the specific molecular processes involved. A lot of research has focused on better understanding tumour biology and the tumour microenvironment (TME) in order to develop novel therapies targeting specific mechanisms that have been implicated in promoting tumour cell survival and progression. Small molecules (chemokines), particularly CXCR4 and its ligand CXCL12, are overexpressed in the tumour microenvironment of most malignant tumours [2]. CXCR4 promotes tumour growth, metastasis, angiogenesis and tumour–cell interaction, and its expression is associated with more aggressive tumour behaviour and poorer prognosis [3]. The CXCR4−CXCL12 axis has been implicated in chemotherapy resistance by providing a protective space for tumour cells in the bone marrow and protecting cancer cells from chemotherapy [4]. Therefore, inhibition of the CXCR4 axis should slow tumour progression and metastasis and improve the chemosensitivity of tumours. Pentixafor is a peptide that targets CXCR4 and a theranostic agent capable of being labelled with Gallium-68 (Ga-68) for positron emission tomography (PET) imaging, and Lutetium-177 (Lu-177) for therapy [5]. Several studies investigating CXCR4 expression in solid tumours have shown variable results and discordance between CXCR4 expression in vivo when compared to immunohistochemistry (in vitro expression of CXCR4) in solid tumours. In this review, the role of ^68^Ga-Pentixafor in the detection of disease in solid tumours will be discussed.

### 1.1. The Tumour Microenvironment

The tumour microenvironment (TME) is an intricate network of the extracellular matrix (ECM), stromal cells and immune cells working together to create a favourable home for tumour cells [6]. The stromal component of the TME is composed of many different cell types, including the cancer-associated fibroblasts, platelets and immune cells such as neutrophils, macrophages, regulatory T cells, myeloid-derived suppressor cells, natural killer cells and mast cells. These crucial parts of the surrounding stroma regulate cancer development, including the avoidance of apoptosis, initiation of angiogenesis, de-regulation of the energy metabolism and resistance to immune detection and destruction, as well as the activation of invasion and metastasis. The TME contributes to tumour aggressiveness and chemoresistance and provides a hostile environment for cancer treatment [7].

Cancer-associated fibroblasts (CAFs) are a group of activated fibroblasts that promote tumour cell growth and proliferation by secreting a large variety of autocrine and paracrine cytokines as well as other tumour-promoting factors [8]. These factors include chemokines, cytokines and growth factors, such as the vascular endothelial growth factor (VEGF), CXCL12 and CXCL14, and interleukins (ILs) IL-6 and IL-17A, which are critical for tumour cell proliferation, angiogenesis, invasion, inflammation, metastasis and drug resistance [9].

Tumour-associated macrophages (TAMs) produce pro-inflammatory cytokines, such as IL-12, IL-23 and tumour necrosis factor-α (TNF-α), and chemokines (CCL-5, CXCL9, CXCL10 and CXCL5) [6]. TAMs also contribute to the proliferation, invasion and metastasis of tumour cells, promote tumour progression and angiogenesis and suppress the T cell antitumor immune response. In most solid cancers such as squamous cell carcinoma of the head and neck (HNSCC) and neuroblastoma the level of TAM invasion correlates with patient outcome [10].

### 1.2. The Role of CXCR-4 in Cancer

Chemokines are small, secreted proteins that organize immunologic and inflammatory processes, such as leukocyte trafficking, adhesion, haematopoiesis and angiogenesis [11]. They are classified into four groups (CXC, CC, C and CX3C) based on the position of the first two cysteines [12]. CXCR4, the receptor of chemokine CXCL12/SDF-1 (stromal cell-derived factor-1), is the most studied chemokine receptor; it has a seven-transmembrane structure with seven helical regions connected by six extra-membrane loops. 

**Figure 1:**
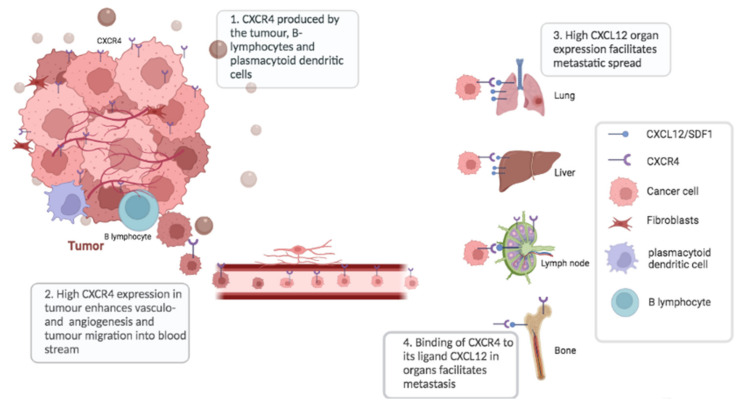
The CXCR4 overexpression in tumours increases angiogenesis, tumour migration and metastasis.

CXCR4 is overexpressed in more than 30 types of human cancer, including uterine carcinosarcoma, lung squamous cell carcinoma, colorectal adenocarcinoma, pancreatic adenocarcinoma, bladder urothelial carcinoma, lung adenocarcinoma, breast cancer, melanoma and lymphoid neoplasms such as diffuse large B-cell lymphoma among others [13]. Its ligand, CXCL12, is expressed in several organs including the lung, liver, skeletal muscle, brain, kidney, heart, skin and bone marrow [14]. CXCR4 activation promotes the migration of cancer cells towards CXCL12-expressing organs such as the lymph nodes, lungs, bone marrow and liver, which leads to the deposition of metastasis in these organs [15]. In addition, CXCR4 also enhances vasculogenesis and hypoxia-driven angiogenesis through the recruitment of CXCR4-positive pro-angiogenic cells, which also promote the spread of cancer cells [16,17]. CXCL12 induces vascular permeability and allows tumour cell extravasation, thus promoting the metastatic process [18] as demonstrated in Figure 1.

CXCR4 is not only expressed by cancer cells themselves, but also by tumour-infiltrating immune cells. Within the tumour microenvironment, the major CXCR4-expressing cells are B lymphocytes and plasmacytoid dendritic cells, both potentially contributing to an immunosuppressive state, which promotes tumour progression [19]. The CXCR4−CXCL12 axis also provides a protective niche for tumour cells in the bone marrow thereby protecting cancer cells from chemotherapy. A few studies have shown the role of CXCR4 expression in predicting sensitivity to cisplatin-based chemotherapy and the role of CXCR4 antagonists in increasing sensitivity to chemotherapy and in improving prognosis [20,21]. CXCR4 expression is higher in cisplatin-resistant cells compared to cisplatin-sensitive cells.

Molecular imaging of CXCR4 using the CXCR4-specific positron emission tomography (PET) tracer, ^68^Ga-labeled cyclopentapeptide, [^68^Ga]CPCR4-2 or ^68^Ga-Pentixafor developed by Demmer et al. [22] has become a well-established method to non-invasively measure CXCR4 expression in vivo. ^68^Ga-Pentixafor attaches to both the CXCR4 and macrophage inhibitory factor (MIF) [23].

The normal biodistribution of ^68^Ga-Pentixafor includes the spleen, kidneys, bone marrow, heart and the liver, which results in undesired tracer accumulation during ^177^Lu- and ^90^Y-Pentixather therapy leading to adverse effects [24,25]. The tumour sink effect, which is characterized by lower tracer accumulation in the normal organs of patients with high tumour burden, has been demonstrated with therapies targeting PSMA and SSTR in prostate cancer and well-differentiated neuroendocrine tumours, respectively [26]. As a result, higher activities can be administered in patients with high tumour burden, resulting in the delivery of a more efficacious ablative dose to the tumours while sparing the non-targeted normal organs. Serfling et al. investigated the potential relationship between tumour burden and radiotracer accumulation in 90 patients with solid cancers using ^68^Ga-Pentixafor. There was no evidence of any tumour sink effect; therefore, the adverse events due to normal tracer biodistribution may limit the administered activity even in patients with a high tumour burden [25]. The authors attributed this difference to the dynamic nature of the CXCR4, which is expressed mainly in the TME compared to PSMA and SSTR, which are more stable and expressed in the tumour cell surface.

Discordance between CXCR4 expression in vivo when compared to immunohistochemistry (in vitro expression of CXCR4) in solid tumours has been reported. For an example, Vag et al. reported that although CXCR4 positivity was seen in vitro in most solid malignancies, including pancreatic carcinoma, laryngeal carcinoma, hepatocellular carcinoma, melanoma, breast cancer, glioblastoma and sarcoma, only low to moderate ^68^Ga-Pentixafor positivity was noted on PET/CT imaging [27]. This may be attributed to the fact that in vitro CXCR4 binds to the cytoplasm, whereas ^68^Ga-Pentixafor binds to membrane-associated chemokine receptors, leading to a significant difference between CXCR4 expression profiles determined by immunohistochemistry and the in vivo quantification of CXCR4 using PET probes.

## 2. CXCR4 in Solid Malignancies

### 2.1. Head and Neck Squamous Cell Carcinoma

Head and neck squamous cell carcinoma (HNSCC) is the sixth most common non-skin cancer and the eighth leading cause of cancer death worldwide; its incidence is rising, with approximately 874,000 new cases per year and a mortality rate of ~50% [28]. The number of deaths is expected to increase from an estimated 300,000 to 595,000 deaths per year worldwide [29]. The survival rate still remains very low and up to 25% of patients develop recurrence or a second cancer within 5 years after diagnosis [30]. Once distant metastasis is diagnosed in HNSCC, the median time to death is estimated at 4 months [31]. The prognosis remains poor due to the disease recurrence and poor response to current treatment regimens [32]. This has led to research into other therapeutic avenues including the use of novel therapies targeting small molecules (chemokines), the most studied one being CXCR4, which is implicated in tumour cell migration and the formation of metastasis in HNSCC [33].

**Figure 2:**
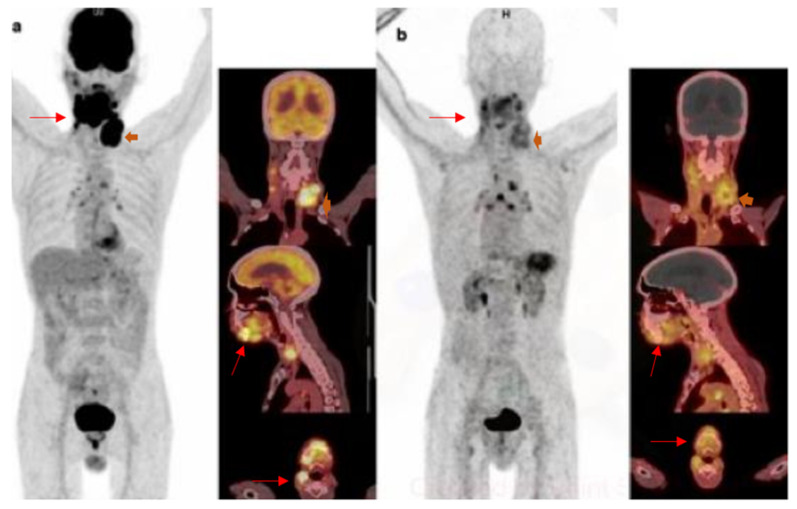
^18^F-FDG and ^68^Ga-Pentixafor images of a 60-year-old female with squamous cell carcinoma of the oral cavity with intense uptake in the primary lesion and left cervical lymph node metastasis on ^18^F-FDG (**a**). There was mild tracer accumulation on the ^68^Ga-Pentixafor PET (**b**). Interestingly, the inflammatory mediastinal lymph nodes appear more intense on the ^68^Ga-Pentixafor PET.

Qiao et al. explored the impact of CXCR4 in tumour invasion in nasopharyngeal cancer. They found significantly higher CXCR4 expression in high grade tumours compared to low grade tumours and the significant suppression of apoptosis [34]. Antagonism of CXCR4/CXCR7-CXCL12 downregulated the expression of the chemokines axis and therefore could be used to control and potentially even cure nasopharyngeal cancer. This was confirmed in a study by Chen et al. using oral cancer cell lines where they found that CXCR4 antagonist, AMD3100, could affect the cell migration and cell invasion of oral cancers [10]. In a recent publication of 690 tumours imaged with ^68^Ga-Pentixafor of which 2 had HNSCC, they demonstrated low tracer uptake with a tumour to a background ratio (TBR) of less than 4 as demonstrated in Figure 2, which shows a patient with squamous cell carcinoma of the oral cavity (subsite tongue) showing intense uptake on the ^18^F-FDG PET/CT but only mild tracer accumulation on the ^68^Ga-Pentixafor PET. However, this is insufficient evidence to make any conclusions, and prospective studies with bigger sample sizes are needed to confirm this finding.

### 2.2. Glioblastoma

Glioblastoma (GBM) is the most common primary malignant brain tumour in adults, and has a poor prognosis with a median survival of only 15 months, despite aggressive treatment [35]. The management of GBM consists of a combination of rescue surgery and temozolomide-based chemoradiation therapy [36]. Delineation of the tumour extent and subsequent treatment planning remains a challenge due to the limited accuracy of MRI. GBM tends to recur and curative treatment does not exist currently; therefore, a theragnostic approach might improve survival rates in patients showing CXCR4 expression. The expression of CXCR4 and CXCL12 mRNAs in glioblastoma increases with tumour grade and is associated with regions of necrosis and angiogenesis [37].

Several studies have demonstrated that CXCR4 expression in glioblastoma is associated with a poorer prognosis and more infiltrative phenotypes. For an example, Jacobs et al. showed that CXCR4 mRNA expression correlates with WHO glioma grade, with a relatively higher expression of CXCR4 in high grade gliomas (obtained from 284 samples), whereas normal brain tissue was CXCR4 mRNA negative [23]. In their study, variable CXCR4 expression was demonstrated in 38% of the cores in glioblastoma cells, and CXCR4 expression was high in only a subset of glioblastomas. This is in contrast with findings by Kamatani et al. where CXCR4 immunoreactivity was observed in 24 of 24 (100%) glioblastomas, 9 of 9 (100%) anaplastic astrocytomas and 10 of 11 (91%) astrocytomas or oligoastrocytomas [37]. Similarly Stevenson et al. demonstrated CXCR4 expression in all five glioblastoma samples [38]. This difference could be due to a smaller sample size in the latter studies.

Jacobs et al. went on to correlate in vitro CXCR4 expression with in vivo ^68^Ga-Pentixafor uptake on PET/CT imaging in seven patients [23]. Five (71%) of the seven patients showed low to moderate tracer uptake, which did not fully correspond with CXCR4 IHC where all patients showed large intra-tumour variation for CXCR4 staining, even within one section. This discrepancy may be attributed to differences in structures between the CXCR4 antibody used for IHC staining and ^68^Ga-Pentixafor or variability in the blood–brain barrier (BBB) crossing between patients to allow for ^68^Ga-Pentixafor uptake. The advantage of ^68^Ga-Pentixafor when compared to ^18^F-FDG is the absence of physiological brain uptake, which increases tumour detection.

With respect to CXCR4-targeted therapies in glioblastoma, the first and most studied CXCR4 antagonist, AMD3100 (Plerixafor), has been reported to inhibit the growth of GBM36 and to reduce glioblastoma stem cell survival in preclinical studies [39]. However, AMD31000 also shows increased cardiotoxicity associated with some other adverse events after long-term use. This has prompted the search for new, safer and selective CXCR4 inhibitors that are suitable as anti-GBM agents. The newly synthesized CXCR4 antagonist, PRX177561, significantly attenuates GBM tumour growth and might augment the effects of antitumour chemotherapy and radiation therapy (RT) [39]. In addition, PRX177561 increases the DFS and OS when administered to mice bearing orthotopic brain xenografts. ^68^Ga-Pentixafor may have a role in selecting patients with glioblastoma who may benefit from therapies targeting CXCR4.

### 2.3. Oesophageal Cancer

Oesophageal cancer (OC) is the eight most common malignant disease and the sixth cause of cancer-related deaths worldwide [36]. The prognosis of OC patients remains unfavourable with 5-year survival rates of only 20 to 36% after curative surgery due to a high potential for lymph node metastasis and vascular invasion. Endoscopic ultrasonography and computed tomography are routine methods of diagnosis of EC patients; however, they have limited usefulness in the early detection of this malignancy, and, therefore, most patients present late with low curative potential [40]. A theragnostic approach might improve survival rates in patients showing CXCR4 expression.

In vitro CXCR4 expression has been shown to correlate with advanced stage, poor differentiation and worse survival in oesophageal cancer. Kaifi et al. demonstrated CXCR4 protein expression in 75 (55%) of 136 oesophageal tumours, and CXCR4 expression was associated with a poor clinical outcome with a median overall survival of 20 months in patients with CXCR4-positive tumours and 76 months for CXCR4-negative tumours [41]. This was confirmed in a meta-analysis of 7 studies looking at 1055 patients with OC, which demonstrated that the overexpression of CXCR4 in OC correlates with reduced OS as well as with tumour depth, lymph node involvement and TNM staging [42]. In a study by Goto et al., 75.6% of the 172 OC specimens stained positive for CXCR4, which significantly correlated with clinical distant metastasis, pathological lymph node metastasis and vascular invasion [43]. Yang et al. performed CXCR4 staining in 101 patients with oesophageal squamous cell carcinoma. Positive expression of CXCR4 was highly expressed in 37.6% (38/101) of OC specimens, and there was no expression in the adjacent normal tissues [44]. 

In 2015, Kobe et al. published the first clinical experience comparing ^68^Ga Pentixafor and ^18^F-FDG in four patients with oesophageal cancer scheduled for radiotherapy [45]. In all patients, FDG was positive for all tumour-suspicious lesions; however, only three out of four patients demonstrated ^68^Ga-Pentixafor uptake in the lesions. Similarly, Linde et al. compared ^68^Ga-Pentixafor and ^18^F-FDG in 10 patients with oesophageal cancer scheduled for radiotherapy. Of the 26 lesions that were counted, 53% of the lesions were positive on both tracers, while 19% of the lesions were FDG positive and Pentixafor negative. Interestingly, Pentixafor revealed more sites of disease than FDG: 7/26 (27%) of the FDG negative lesions were Pentixafor positive [46]. CXCR4 expression was confirmed immunohistochemically in four lesions of the seven patients, where histopathologic correlation was performed. ^68^Ga-Pentixafor provided additional information that altered patient management, which resulted in the adjustment of the irradiation field based on a Pentixafor-positive and FDG-negative lesion that was detected outside the radiotherapy field.

### 2.4. Neuroendocrine Tumours

The management of neuroendocrine carcinomas is challenging because most of them present with metastasis and respond poorly to chemotherapy [47]. There has been interest in identifying other treatment options for patients with poorly differentiated neuroendocrine tumours (NETs) including CXCR4-targeted therapies as strong receptor expression on the tumour cell surface would pave the way for CXCR4-targeted radionuclide therapy in somatostatin receptor (SSTR)-negative patients, given the limited treatment options in de-differentiated NETs.

To this end, Werner et al. compared ^68^Ga-Pentixafor, ^68^Ga-DOTA-D-Phe-Tyr3-octreotide (^68^Ga-DOTATOC) and ^18^F-fluorodeoxyglucose (^18^F-FDG) in 12 patients with gastroenteropancreatic NET [48]. ^68^Ga-Pentixafor was the least sensitive of the three tracers and detected tumour lesions in only 50% of the patients, whereas ^18^F-FDG was positive in 85% and ^68^Ga-DOTATOC in 92% patients, respectively. As expected, ^68^Ga-DOTATOC was more sensitive than Pentixafor in well-differentiated NET (G1 and G2 NET), and CXCR4-directed PET was negative in all G1 NET. Conversely, 50% of G2 and 80% of G3 NET patients demonstrated ^68^Ga-Pentixafor-positive tumour lesions, which is in keeping with higher CXCR4 expression in more aggressive higher grade tumours [48]. ^68^Ga-Pentixafor uptake was collaborated by histology in 8/9 (89%) of the participants, and the site of histological biopsy was still present for 9/12 subjects. Similarly, Weich et al. investigated 11 treatment-naïve patients with histologically proven neuroendocrine carcinomas (NEC), who underwent ^18^F-FDG and CXCR4-directed PET/CT for staging and therapy planning [47]. CXCR4-directed imaging was less sensitive compared to FDG, and ^68^Ga-Pentixafor visualized tumour lesions in 10/11 patients, while ^18^F-FDG revealed sites of disease in all 11 patients. ^18^F-FDG PET/CT detected significantly more tumour lesions (102 vs. 42 of the total 107 lesions). Weak to moderate CXCR4 expression could be corroborated by IHC in 10/11 cases [47]. From these studies, it appears that there may be a role for CXCR4-directed imaging in patients with higher grades (G2 and G3) of neuroendocrine tumours. Despite its inferiority to FDG as demonstrated by Weich et al. [47], ^68^Ga-Pentixafor has a role in higher grade tumours and offers the potential advantage of targeted radionuclide therapy in NECs with no SSTR expression.

### 2.5. Lung Cancer

Small cell lung cancer (SCLC) represents 15% of all lung cancers and is characterized by a rapid doubling time, the early development of metastases and a poor prognosis. Only one-third of patients are diagnosed with localized disease, and, as a result, treatment strategies have focused on systemic therapy [49]. SCLC is highly responsive to both chemotherapy and radiotherapy initially; however, it has a high recurrence rate within months of treatment due to the development of multidrug resistance (MDR).

Unfortunately, there are limited therapeutic options for relapsed SCLC to date, and new approaches are urgently needed to overcome the MDR of SCLC, which includes the use of CXCR4 antagonists. A few studies have investigated CXCR4 expression in SCLC to see if these patients could potentially benefit from therapies targeting CXCR4. Emerging research shows SCLCs do expresses CXCR4 in vitro, and CXCR4 expression correlates with poor overall survival. Kaemmerer et al. evaluated 34 small cell lung cancer samples by immunohistochemistry, and, in their study, CXCR4 predominantly localized at the plasma membrane of the tumour cells, which was correlated with clinical data and overall patient survival [50].

In a case report, Watts et al. demonstrated higher tracer accumulation on ^68^Ga-Pentixafor compared to ^18^F-FDG in SCLC [51]. Furthermore, SCLC showed higher CXCR4 expression in vitro and a higher uptake (SUVmax = 13.2) of ^68^Ga-Pentixafor (maximum fluorescence intensity MFI = 142.0) compared to NSCLC (MFI = 120; SUVmax = 8.8). This is in line with the higher aggressiveness of SCLC compared to NSCLC. Lapa et al. evaluated 10 patients with therapy-naïve, 3/10, or pretreated, 7/10, lung cancer using ^68^Ga-Pentixafor PET/CT. Nine (9) of the 10 patients had SCLC and one had large cell neuroendocrine carcinoma of the lung. A comparison with ^18^F-FDG was performed in six patients and a comparison with ^68^Ga-DOTATOC was performed in five patients. ^68^Ga-Pentixafor PET was positive in 80% of the patients and revealed more lesions with significantly higher tumour to background ratios than ^68^Ga-DOTATOC-PET. Two patients who were ^18^F-FDG PET positive were negative on ^68^Ga-Pentixafor-PET. Interestingly, ^68^Ga-Pentixafor-detected lesions were not seen on ^18^F-FDG PET. Furthermore, the CXCR4 expression of tumour lesions could be confirmed by immunohistochemistry [49].

Similarly, in a study by Li et al., CXCR4 and CXCL12 were expressed in 97.6% and 78.0% of the six mesothelioma tissue samples, respectively [52]. Conversely, Lapa et al. also compared ^18^F-FDG and ^68^Ga-Pentixafor in six patients with pleural mesothelioma as well as immunohistochemistry obtained from biopsy or surgery, and, in their study, none of the six patients presented ^68^Ga-Pentixafor-positive lesions, whereas ^18^F-FDG PET revealed positive lesions in all patients [53]. On immunohistochemistry, no significant CXCR4 surface expression was identified in the samples analysed. 

CXCR4-targeted therapies in SCLC show promise in preclinical studies; a study by Otani et al. showed a significant decrease in the number and size of lung metastases in mice with CXCR4-expressing SCLC treated with the peptide-based inhibitor of CXCR4, TF14016 [54]. The heterogenous CXCR4 expression shown in these studies shows that CXCR4-targeted imaging cannot replace ^18^F-FDG in lung cancer; however, CXCR4-targeted imaging with ^68^Ga-Pentixafor can be used to select patients who may benefit from these therapies and to monitor treatment response. Moreover, CXCR4-targeted imaging may alter patient management in a select group of patients where it shows more lesions than FDG, but this has to be confirmed in a larger cohort.

### 2.6. Osteosarcoma

Osteosarcoma (OS) is the most common primary malignant tumour of bone with a 5-year survival rate of 25–30% in metastatic OS, which occur in 30% of cases [28]. Although pre- and postoperative chemotherapy has improved prognosis in osteosarcoma (OS), treatment failure is often seen due to high toxicity and resistance to chemotherapy. Recent research has been directed into better understanding tumour biology in order to identify new biomarkers that can predict chemoresistance and therapeutic strategies that may increase drug sensitivity and better control localized and metastatic disease [55].

Gong et al. evaluated 63 tumour samples with CXCR4 immunohistochemistry, and the patients were followed up for 36 months to evaluate for tumour metastasis and patient survival. In that study, 68.5% of tumours showed CXCR4 expression, and of the 45 patients who developed distant metastasis, 33 (73%) had positive expression of CXCR4. The median metastasis-free survival and overall survival was higher in the CXCR4-negative group compared to the CXCR4-positive group [56]. Similarly, Pollino et al. assessed CXCR4 expression in 48 primary osteosarcoma samples compared to 10 healthy bone tissues samples. They demonstrated that in osteosarcoma, CXCR4 expression was directly proportional to the histological grade of the tumour and aggressiveness [55]. Thus far, there is insufficient evidence on the role of ^68^Ga-Pentixafor in osteosarcoma; however, from the published data on immunohistochemistry, this tracer may have a role in prognosis and selecting patients who may benefit from therapies targeting CXCR4.

### 2.7. Gynaecological Malignancies

#### 2.7.1. Vulva Cancer

Vulvar squamous cell carcinoma (VSCC) is a rare disease accounting for about 4% of all gynaecological malignancies; however, the incidence is increasing in HIV-infected patients where VSCC is seen in younger patients [57]. VSCC spreads predominantly via lymphatic spread, and the presence of inguinal lymph nodes is an independent predictor of survival. Patients with histologically negative inguinal lymph nodes (LNs) have survival rates of 90% vs. 50% for those with histologically positive inguinal LNs [58].

In a study by Shiozaki et al., in vitro CXCR4 expression was seen in 68% of the 22 tested VSCC samples [59]. CXCR4 expression correlated with more aggressive disease, and higher expression was seen in FIGO stages (III-IV) compared to lower stages (I-II); however, this correlation was not statistically significant (*p* = 0.08). In addition, the expression rate of CXCR4 in lymph node-positive metastatic disease was very high and CXCR4 expression was associated with poor disease prognosis [59]. Similarly, Rusetska et al. also evaluated CXCR4 expression on tumour and lymph node tissue samples obtained from 46 patients with VSCC as well as 51 patients with premalignant vulvar lesions. CXCR4 overexpression was noted in VSCC samples and none in vulvar precancers; additionally, 98% of the metastatic LNs of patients with VSCC expressed CXCR4 [58]. Again, in vulvar carcinoma, CXCR4-targeted imaging could potentially be used to prognosticate patients as well as to select patients who may benefit from therapies targeting CXCR4. Furthermore, ^68^Ga-Pentixafor may be able to differentiate premalignant lesions from carcinoma of the vulva.

#### 2.7.2. Cervical Cancer

**Figure 3:**
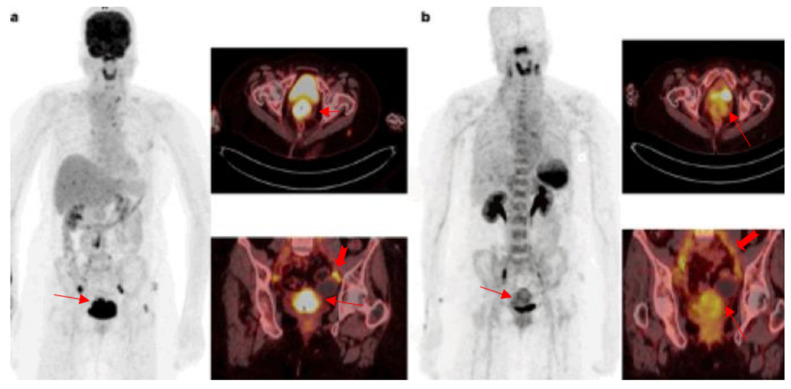
^18^F-FDG and ^68^Ga-Pentixafor images of a 36-year-old female patent with squamous cell carcinoma of the cervix with intense uptake in the primary lesion and pelvic lymph node metastasis on ^18^F-FDG (**a**). There is moderate heterogenous tracer accumulation on the ^68^Ga-Pentixafor PET and very low uptake on the metastatic lymph node (**b**).

Cervical cancer is the second most prevalent cancer in the world after breast cancer [60] and represents 6.6% of all cancers of females, with an estimated 570,000 new cases in 2018. In low- and middle-income countries, cervical cancer has a dismal prognosis with an average death rate of 90% [61]. Better treatment strategies are urgently needed since this condition has a high rate of disease recurrence and/or progression of approximately 75% despite the use of available treatment strategies such as surgery, radiotherapy and chemotherapy. Dai et al. evaluated CXCR4 expression in 57 tissue samples consisting of 9 normal cervical tissue samples and 48 cervical cancer samples using immunohistochemistry stains. Of the 48 cancer tissues, 31 (64.58%) were CXCR4 positive, and, in addition, CXCR4 expression correlated with more aggressive histology; that is, it was significantly higher in patients with squamous cell carcinomas and lymph node metastasis [62]. This was confirmed by Schrevel et al. who found that high CXCR4/CXCR7 co-expression independently correlated with shorter disease-specific survival and it was positively associated with larger tumour size and lymph nodes metastasis [18]. Although there are currently no studies demonstrating the role of ^68^Ga-Pentixafor in cervical cancer, it appears that CXCR4-targeted imaging could potentially be used to prognosticate patients as well as to select patients who may benefit from therapies targeting CXCR4. Figure 3 shows intense ^18^F-FDG uptake in the cervical carcinoma primary and metastatic left pelvic lymph node (a) and moderate accumulation of ^68^Ga-Pentixafor in the cervix and very mild uptake in the metastatic lymph node.

#### 2.7.3. Ovarian Cancer

Ovarian cancer is the seventh most common cancer in women globally, and it contributed to approximately 300,000 new cases in 2018 [35]. Ovarian cancer is the main cause of death in gynaecological malignancies. The high mortality of ovarian cancer patients can be attributed to chemotherapy resistance and extensive intraperitoneal metastasis. Although ovarian cancer is one of the most chemo-sensitive malignancies, the prognosis is still poor, even after radical surgical tumour debulking and subsequent chemotherapy [63]. This is believed to be due to the lack of cancer-specific symptoms during the early stages of disease, with ovarian cancer patients usually diagnosed at a late stage with advanced disease. It is estimated that in the USA 80% of ovarian cancer cases have regional or distant cancer spread at the time of cancer diagnosis, and the five-year relative survival of ovarian cancer is less than 50% [64]. There is evidence that CXCR4 is expressed in ovarian cancer primary tumours and its ligand CXCL12 is expressed in the ascitic fluid [64,65]. In a meta-analysis of seven studies (729 patients), it was shown that CXCR4 expression detected on immunohistochemistry staining correlated with more advanced disease and poorer overall survival [64]. There have not been any studies thus far investigating the role of ^68^Ga-Pentixafor PET/CT in ovarian cancer.

### 2.8. Breast Cancer

Breast cancer is the most common cancer among women with an estimated 2.3 million new cases diagnosed globally each year; more than 2 million new breast cancer cases were diagnosed in 2020 [66]. Its incidence and death rate have increased over the last three decades due to the change in risk factor profiles. Metastatic breast cancer has a high mortality, especially for late-stage patients [67]. Despite early detection and improved therapeutic regimens, drug resistance, relapse and metastasis still occur and result in significant reduction in the survival of breast cancer patients [68]. CXCR4 receptor and its ligand CXCL12 play a crucial role in the metastasis of various cancer types including breast cancer [69].

The overexpression of CXCR4 on immunohistochemistry has been shown to be associated with poorer prognosis in breast cancer patients. Muller et al. reported that patients with high levels of CXCR4 had more extensive metastasis to lymph nodes compared to those with low levels of CXCR4 [70]. CXCR4 also promotes breast cancer metastasis to organs (bone, liver and lung) where there is an abundance of its ligand, SDF-1. A meta-analysis by Zhang et al. included 13 eligible studies consisting of 3865 participants and showed that CXCR4 overexpression was associated with lymph node infiltration, distant metastasis and significantly reduced disease free survival (DFS) and overall survival (OS) [71]. This was confirmed in a meta-analysis of 15 studies by Xu et al., which included 3104 patients, that showed that the OS and DFS were lower in breast cancer patients with high levels of CXCR4 expression compared to those with low levels of CXCR4 expression [72]. 

With respect to ^68^Ga-Pentixafor PET/CT imaging in breast cancer, tracer accumulation is associated with poorer prognosis; however, there is no statistically significant correlation between tracer uptake and molecular subtypes of breast cancer or Ki67% [19]. In a retrospective analysis, Vag et al. evaluated 18 patients with breast cancer with ^68^Ga Pentixafor PET/CT, and eight patients additionally underwent ^18^F-FDG PET/CT for staging purposes. Nine (69%) of 13 primary breast cancers were visually detectable on ^68^Ga-Pentixafor PET images; furthermore, metastases of recurrent breast cancer and unknown primary cancer were visually detectable in all five cases, with a mean SUVmax of 3.5. In another study comparing ^68^Ga-Pentixafor with ^18^F-FDG in a heterogenous group of patients of which two had metastatic breast cancer, ^68^Ga-Pentixafor was inferior to FDG and detected four of the seven metastatic lesions and both the primary tumours seen on ^18^F-FDG PET/CT [27]. The SUVmax obtained during ^18^F-FDG PET was higher in all cases compared to CXCR4-targeted PET. This means that ^68^Ga-Pentixafor could help guide therapy by identifying tumours that would respond to therapies targeting the CXCR4/CXCL12 axis; however it cannot replace ^18^F-FDG PET/CT in the staging of breast cancer. 

A few CXCR4 antagonists have been investigated in breast cancer models and have shown promising results [73]. These CXCR4 antagonists block the CXCR4 receptor binding to its ligand SDF-1 and could be potential anticancer agents for the treatment of breast cancer [74].

### 2.9. Prostate Cancer

Prostate cancer is the second most common cancer diagnosed in men and the fifth leading cause of death worldwide, and its incidence is increasing [75]. Despite advances in the treatment of localized disease, many patients progress to metastatic castration-resistant prostate cancer, which is associated with poor survival and significant morbidity [76]. There has been a huge breakthrough in the treatment of prostate cancer with hormonal therapies and targeted radionuclide therapies such as ^177^Lu-PSMA and ^225^Ac-PSMA, but these are not yet widely available. In prostate cancer patients, CXCR4 expression is significantly associated with a more aggressive disease, the presence of metastasis and poorer cancer-specific survival. 

Schwarzenböck et al. compared ^68^Ga-Pentixafor, ^18^F FDG and functional MRI in 19 xenograft mouse models and tumour uptake was lower compared to ^18^F-FDG. Bladder activity was high on ^68^Ga-Pentixafor due to the predominant urinary excretion of the tracer, which makes delineation of bladder infiltration challenging, and poses the same problem encountered with ^68^Ga-PSMA and ^18^F-FDG where bladder and ureteric activity may interfere with the interpretation of locoregional lymph node and prostatic tracer accumulation. Liver uptake of ^68^Ga-Pentixafor was comparable, whereas kidney uptake of ^68^Ga-Pentixafor was slightly less than ^18^F FDG [77]. These studies show that ^68^Ga-Pentixafor does accumulate in prostate cancer; however, studies comparing this tracer to ^68^Ga-PSMA are needed to see if CXCR4 targeted imaging and therapy can replace PSMA, especially with the suboptimal response of ^177^Lu-PSMA in patients with bone metastasis. The advantage of ^177^Lu-Pentixather over PSMA might be that ^177^Lu-Pentixather can be directed at tumour cells and neovasculature when CXCR4 is present, unlike PSMA which only targets the neovasculature [23].

### 2.10. Vestibular Schwannoma

Vestibular schwannomas (VS) are benign nerve sheath tumours of the vestibulocochlear nerve [78]. They can be sporadic or associated with neurofibromatosis type 2 (NF 2). NF 2 tumours have a propensity to recur and to grow more rapidly, and adhere to the cranial nerves and the brainstem more commonly compared to sporadic schwannomas [79]. They are harder to treat with surgery and, thus, alternative treatment options are being investigated including CXCR4 antagonists.

Breun et al. studied CXCR4 expression in 60 vestibular schwannoma (vs) samples and compared them with nerves from autopsies, which served as controls. They found higher expression of CXCR4 in vs compared to the controls: CXCR4 mRNA levels were 4.6-fold higher in vs versus the controls [80]. Furthermore, even though CXCR4 expression was not associated with tumour size, it showed a correlation with tumour invasiveness; that is, higher CXCR4 expression was seen in vs patients with severe hearing loss and deafness.

In another study, four patients with six vs lesions were evaluated with ^68^Ga-Pentixafor PET/CT. Even though the sample size was very small, they demonstrated the ability of ^68^Ga-Pentixafor PET/CT to detect all schwannomas with sufficient tumour to background and tumour to blood pool ratios that matched with membranous CXCR4 expression as assessed by immunohistochemistry [78]. Therefore ^68^Ga-Pentixafor PET/CT could potentially be used to select patients who would benefit from targeted therapies with a CXCR4 antagonist.

### 2.11. Adrenocortical Carcinoma

Adrenocortical carcinoma (ACC) is a rare and aggressive disease with limited therapeutic options in advanced disease, which commonly recurs despite complete resection [81]. Mitotane in combination with chemotherapy is used as a first-line systemic therapy in patients with metastatic disease; however, recurrence is seen in approximately 50%, even after mitotane therapy, and all available systemic therapies are only palliative. Disease response is achieved in only a minority of patients; therefore, other treatment options are being investigated including CXCR4 inhibitors. 

Chifu et al. evaluated the in vitro expression of CXCR4 in 18 ACC tumour samples and found a strong membrane expression of CXCR4 in 50% of ACC samples [82]. Interestingly, strong cytoplasmic CXCR4 staining was more frequent among samples derived from metastases compared to primary tumours and local recurrences. Moreover, CXCR4 membrane staining positively correlated with the proliferation index Ki67. Similarly, Heinze et al. observed CXCR4 membrane staining in 83% of the 21 tumour tissue sections of primary tumours and metastases of ACC, and there was shorter disease free survival in tumours with high CXCR4 expression [83]. There was no difference in progression-free or overall survival observed between patients with strong and weak staining intensities for CXCR4.

Subsequent to that, Buck al. imaged six ACC patients with ^68^Ga-Pentixafor and found high tracer accumulation in ACC with good target-to-background ratios [84]. Bluemel et al. compared ^68^Ga-Pentixafor with ^18^F-FDG in 22 patients with ACC, and the two tracers demonstrated comparable findings in seven (32%) patients [85]. In nine patients (41%), ^18^F FDG identified more lesions with a visually higher uptake compared to ^68^Ga-Pentixafor, whereas ^68^Ga-Pentixafor identified more metastatic lesions than ^18^F-FDG in two patients (9%). In their study, 12 out of 22 patients (54%) were rated as suitable and 3 patients (14%) as potentially suitable for targeted radionuclide therapy with [^177^Lu]/[^90^Y]-labelled Pentixather. Similarly, in another study of thirty patients with histologically proven advanced metastatic ACC who underwent ^18^F-FDG PET/CT and ^68^Ga-Pentixafor to evaluate suitability for CXCR4-targeted therapy, ^18^F-FDG PET/CT provided a superior detection rate than ^68^Ga-Pentixafor PET/CT with a visually higher uptake in 43% of patients [86]. Again, ^68^Ga-Pentixafor PET identified more lesions compared with ^18^F-FDG PET in 2/30 patients (7%) [86]. Therefore, ^68^Ga-Pentixafor imaging could potentially be used to identify adrenocortical carcinoma patients who would benefit from CXCR4-targeted therapies as not all lesions express CXCR4.

### 2.12. Colorectal Cancer

Colorectal cancer (CRC) is the third leading cause of cancer-related death worldwide [28]. Unfortunately, about 30% of patients present with metastatic disease involving predominantly the liver, lungs and lymph nodes. The treatment of advanced diseases is multimodal comprising radiotherapy, surgery, and/or chemotherapy and targeted therapies. CRC cells that express high CXCR4 levels have been shown to be more invasive and are associated with worse long term survival, and expression has been reported to be higher in colorectal liver metastases than the primary tumour [87].

Ottaiano et al. evaluated CXCR4 expression in 78 CRC primary colon cancer samples by immunohistochemistry. CXCR4 expression on the primary tumour was an independent prognostic factor and correlated with the response to first-line chemotherapy in metastatic CRC patients [88]. Furthermore, in a meta-analysis, Jiang et al. confirmed that high CXCR4 expression in oesophagus, gastric and colorectal cancer predicted a worse prognosis [89].

In a pilot study, Ahern et al. compared ^68^Ga-Pentixafor with ^18^F-FDG PET/CT imaging in eight patients with colorectal adenocarcinoma. In that study, ^68^Ga-Pentixafor showed low to moderate tracer accumulation, and none of the patients qualified for targeted radionuclide therapy [90]. Furthermore, ^68^Ga-Pentixafor was inferior to ^18^F-FDG; therefore, ^68^Ga-Pentixafor cannot replace but remains complementary to ^18^F-FDG PET/CT, and its primary role may be in the selection of patients who will benefit from targeted therapy using a CXCR4 antagonist. However, this was a very small sample size, and more research is needed in order to draw reasonable conclusions.

### 2.13. Neuroblastoma

Neuroblastoma is the most commonly diagnosed extra-cranial solid tumour of infancy and childhood accounting for 8–10% of all paediatric (0–14 years) tumours, and for approximately 15% of cancer-related paediatric mortality [91]. South Africa has a higher-than-average number of patients with high-risk tumours (75.6%), mainly because of advanced disease (70%) and a 54% MYCN amplification of tumours [92]. The prognosis for neuroblastoma remains poor; therefore, there is a need for the development of effective therapies targeting the tumour microenvironment, which creates an unfavourable environment for most of the current treatment strategies [93]. CXCR4 expression is associated with a highly aggressive undifferentiated histopathologic type [13] and poor prognosis in neuroblastoma tumours [94]. CXCR4 favours neuroblastoma metastasis to the liver as well as the lungs and increased bone marrow invasion [7]. ^68^Ga-Pentixafor may have a role in identifying patients with a poorer prognosis and those who are candidates for therapies targeting CXCR4.

### 2.14. Hepatocellular Carcinoma

Hepatocellular carcinoma (HCC) is the sixth most common cancer in the world with a rising incidence, and it is the fourth leading cause of cancer death globally [95]. HCC remains asymptomatic until it is very advanced, which makes early detection important in reducing HCC-related mortality. The management of HCC depends on the stage of the tumour and the severity of the underlying liver disease, with resection and transplant still the best curative options for early-stage disease [96]. The role of cytotoxic chemotherapy is limited in HCC. Recent advances in locoregional therapy and molecular targeted systemic therapy, such as small molecule tyrosine kinase inhibitors (TKIs) and immune check point inhibitors, have changed the management of intermediate and advanced HCC. However, these are not readily available due to the high cost, and TKIs are also limited by their side effects.

CXCR4 is overexpressed in 88.33% (65/78) of HCC, where it plays a significant role in the metastasis of HCC by promoting the migration of tumour cells [97]. In a study by Werner et al. comparing ^68^Ga-Pentixafor and ^18^F-FDG in a heterogenous group of solid tumours, of which three were HCCs, ^68^Ga-Pentixafor detected the tumour in only one of the three HCC patients. [98]. This was confirmed by Vag et al. where CXCR4 was detected in vivo in one of the two patients imaged with ^68^Ga-Pentixafor PET/MRI [27]. Unfortunately, the sample size was too small to draw any meaningful conclusions except to say that non-invasive detection of CXCR4 expression with ^68^Ga-Pentixafor PET/CT could be used to select patients who would benefit from therapies targeting CXCR4.

### 2.15. Melanoma

Malignant melanoma is one of the most aggressive neoplasms with frequent radio- and chemotherapy resistance. Malignant melanoma has an incidence of 15–25 per 100,000 individuals, which is increasing faster than any other cancers [28]. Metastatic malignant melanoma has a poor prognosis, with a 15–20% five-year survival rate [99]. Chemotherapy and radiation therapy are not considered to be good options for the treatment of metastatic melanoma because of treatment resistance [100].

Melanoma has a high immunogenicity and is infiltrated with various immune cells including chemokines. CXCR4 enhances the proliferation and survival of melanoma cells by increasing the number of tumour blood vessels. In vivo CXCR4 overexpression in melanoma has been reported [101] and has been found to be higher in metastasis than primary tumours, and, in addition, it is associated with a higher tumour stage [99]. In a meta-analysis of 13 studies investigating the CXCR4 expression in the tumour samples of 656 patients with malignant melanoma, it was found that the high expression of CXCR4 is associated with ulceration, increased tumour thickness and lymph node metastasis [102]. With respect to imaging, in a study by Vag et al. comparing ^68^Ga-Pentixafor with ^18^F FDG in a heterogenous group of patients of whom two had malignant melanoma, ^68^Ga-Pentixafor detected all four of the four metastatic lesions, as did ^18^FDG PET. On ^18^F FDG PET/CT, the SUVmax was slightly higher than on ^68^Ga-Pentixafor [27]. Thus, CXCR4-targeted imaging cannot replace ^18^F-FDG but may have a role in the selection of potential candidates for therapy with CXCR4 antagonists.

### 2.16. Cholangiocarcinoma

Cholangiocarcinoma (CCA) is the second most common malignancy of the liver after hepatocellular carcinoma (HCC). It is aggressive and fatal with a median survival of less than 24 months [103]. While surgery is the most effective treatment for resectable CCA [104], unfortunately the clinical symptoms of CCA are non-specific and early diagnosis is often difficult. As a result, the majority of CCA patients are diagnosed at an advanced stage, at which therapeutic options are limited [105]. Thus, CCA patients are offered palliative care with an overall 5-year survival rate of <10%. Due to the lack of effective medical treatment, molecular imaging is essential to guide the discovery of targeted therapies. CXCR4-targeted imaging provides an alternative for possible therapeutic options with a CXCR4 antagonist; however, there is limited evidence on the CXCR4 expression of CCA.

Leelawat et al. demonstrated the upregulation of CXCR4 in cholangiocarcinoma, which is associated with poorer prognosis [106]. This was confirmed by Zhao et al. who investigated the functional role of CXCR4 in the progression and metastasis in 122 patients with intrahepatic cholangiocarcinoma and found CXCR4 expression in the cytoplasm of most IHCC cells but not in the adjacent non-tumorous tissues [107]. Sixty (49%) of the patients had high CXCR4 expression and 62 had low CXCR4 expression and high CXCR4 expression, which were associated with metastasis and a poor clinical outcome of IHCC. Tan et al. demonstrated a positive correlation between the expression of CXCR4 and lymph node metastasis [108].

Werner et al. imaged 19 patients with solid tumours of which 3 had cholangiocarcinoma, and they demonstrated a high uptake of ^68^Ga-Pentixafor in CCC in both the primary and metastatic lesions in all the patients with CCC [98]. In fact, one of the patients with cholangiocarcinoma demonstrated the highest level of radiotracer uptake (SUVmax, 16.0; TBR, 7.4). This finding needs to be confirmed in a bigger sample but shows promise.

### 2.17. Pancreatic Ductal Adenocarcinoma

Pancreatic ductal adenocarcinoma (PDAC) is one of the most aggressive tumour types and the fourth leading cause of tumour-related mortality in developed nations [28]. There are no specific tumour markers for PDAC and symptoms are often not present during early disease; therefore, most patients present with advanced, inoperable disease [109]. Current chemotherapeutic drugs that are used for advanced disease stages show dismal results and toxicity seems to be a major problem. Therefore, there is a need to find safe and more effective therapies that can target PDAC. To this end, a lot of work has been carried out to investigate CXCR4 expression in PDAC with a view to establish whether a CXCR4 antagonist can be used in this condition.

In a meta-analysis by Ding et al. of 11 studies (1439 patients), the pooled findings demonstrate that CXCR4 expression is present in PDAC, and it positively correlated with tumour grade, tumour stage, lymph node invasion and distant metastasis [110]. Furthermore, CXCR4 overexpression was a poor prognostic indicator in patients with PDAC. This was confirmed by Krieg et al. who demonstrated that CXCR4 expression levels were related to metastatic disease and overall survival in patients with PDAC in their meta-analysis of nine studies (1183 patients) [109].

In the study by Werner et al. evaluating the uptake of ^68^Ga-Pentixafor in solid tumours, 4/19 patients reported on had pancreatic ductal adenocarcinoma, which demonstrated mild tracer accumulation with an average TBR of 2.92 (2.07–3.72) [98]. In a recent study by Buck et al. looking at ^68^Ga-Pentixafor uptake in 690 patients with solid and haematologic tumours, 8/535 scans belonged to patients with pancreatic adenocarcinoma and there was moderate tracer accumulation with a TBR of >4 (average 4–6) [84]. Based on these results, ^68^Ga-Pentixafor could potentially have a role in the prognosis of pancreatic cancer and selecting patients who may benefit from therapies targeting CXCR4; however, more research is needed to confirm this.

### 2.18. Renal Cell Carcinoma

Renal cell carcinoma (RCC) is the most lethal of the common urological cancers with a 5-year relative survival of 75.2% in the US [111]. Approximately a third of the patients have metastatic RCC at the time of presentation, and another third who present with local disease will eventually develop disease recurrence and metastases with a median survival of less than one year. RCC is a highly vascularized tumour, in which the von Hippel Lindau tumour suppressor gene is frequently inactivated, leading to the overexpression of the hypoxia-inducible factor (HIF)-2α oncoprotein and CXCR4 overexpression [112].

In a meta-analysis of fourteen studies with 1203 participants by Si et al., they showed that CXCR4 expression is significantly higher in patients with metastatic RCC than in patients with non-metastatic RCC, and CXCR4 expression is significantly associated with poorer prognosis [113]. Pan et al. compared CXCR4 expression in 21 patients with metastatic RCC with three normal volunteer control subjects and they found high CXCR4 expression in the circulating cells of the RCC patients [114].

Werner et al. imaged 19 patients with solid tumours of whom 1 had renal cell carcinoma, and they demonstrated very low uptake and TBR of ^68^Ga-Pentixafor in RCC [98]. Thus, CXCR4 expression could be a potential biomarker of prognosis and a drug target for personalized treatments for patients with RCC [113], but the evidence is still lacking and more studies are needed to determine whether ^68^Ga-Pentixafor can reliably detect CXCR4 expression in vivo in patients with RCC.

### 2.19. Gastric Cancer

Gastric cancer (GC) is one of the most common malignancies globally, and it is the fourth leading cause of cancer-related death [115]. There has been a decline in the incidence and mortality over the past 5 decades; however, gastric cancer still remains one of the leading causes of cancer-related death [116]. The incidence rates for gastric cancer are 2-fold to 3-fold higher for men than women with a mortality rate of 75% in most countries. CXCR4 overexpression has been reported in gastric adenocarcinoma [117], and its expression is associated with high tumour stage, poorer prognosis [118], larger tumour size, lymphatic invasion [87] and carcinomatosis [119].

Thus far, there are no published reports on ^68^Ga-Pentixafor accumulation in gastric cancer; however, there is evidence from preclinical studies using GC cell lines that there is CXCR4 overexpression.

Table 1 summarises the available data on CXCR4 and the potential applications of ^68^Ga Pentixafor in solid tumours.

## 3. Future Direction

An ^18^F-radiolabelled cyclam-based small molecule radiotracer, [^18^F]MCFB, for imaging CXCR4 expression has also been investigated in preclinical studies and shows promise as a molecular probe for CXCR4 expression [16]. ^18^F has superior properties to ^68^Ga such as a low positron energy, better image quality and adequate half-life (109.7 min). In addition, a ^99m^Tc-labelled agent, [^99m^Tc]TcAMD3465, has been explored for imaging CXCR4 expression in preclinical studies and has shown a significantly high tumour to background ratio; this will be useful in developing countries where PET imaging is not widely available.

### 3.1. Therapies Targeting CXCR4

The CXCL12/CXCR4 axis has been identified as a target for drug development in oncology due to its critical role in promoting and maintaining cancer stem cells [47]. CXCR4 is present on both the neovasculature and the tumour cells. Blockade of CXCR4 is thought to inhibit all the steps necessary for the development of metastatic foci: not only tumour cell infiltration into CXCL12-producing predestination organs, but also adhesion, migration, invasion and tumour angiogenesis [54]. Cancer stem cells, which are believed to represent a drug-resistant cell population that can survive even chemotherapy, overexpress CXCR4, which correlates with tumour aggressiveness and metastatic potential [27]. Therefore, targeting CXCR4 slows tumour growth, reduces angiogenesis as well as the expression of VEGF [9] and increases the sensitivity of malignancies to chemotherapy, leading to apoptosis [4,120]. Wang et al. demonstrated that the spread and metastasis of oesophageal cancer stem cells could be inhibited by the blockage of CXCR4 [2].

The CXCR4 antagonist Plerixafor (AMD3100) is the first and most studied CXCR4 antagonist, originally developed to block the entry of HIV-1 viruses into T cells through CXCR4 and induce progenitor/stem cell mobilization from the bone marrow for HIV treatment. It also inhibits the binding of CXCL12 to CXCR4 and inhibits cell proliferation, migration and invasion. Plerixafor was approved by the FDA in 2008 for non-Hodgkin’s lymphoma and multiple myeloma [120]. However it is limited by cardiotoxicity, and this has led to development of other CXCR4 antagonists [13]. Another CXCR4 antagonist, BPRCX807, has been shown to be effective in inhibiting migration and suppressing metastasis in hepatocellular carcinoma [121,122]. In addition, GST-NT21MP, which is a synthetic 21-mer peptide antagonist of CXCR4 (NT21MP) derived from the viral macrophage inflammatory protein II, was found to block the CXCR4 pathway, thus decreasing SDF-1-induced cell growth, adhesion and migration capacities in breast cancer cell lines [123]. The newly synthesized CXCR4 antagonist, PRX177561, significantly attenuates GBM tumour growth and might augment the effects of antitumour chemotherapy and RT. In addition, PRX177561 increases the DFS and OS when administered to mice bearing orthotopic brain xenografts [39].

### 3.2. CXCR4-Targeted Radionuclide Therapy

Initial reports on the role of CXCR4-targeted radionuclide therapies in haematological malignancies using ^177^Lu- and ^90^Y-Pentixather have shown promising results, and the treatment was tolerated well with no acute adverse events [29,30,31]. Unfortunately, because the CXCR4-targeted therapies do not only eradicate the CXCR4-expressing malignant cells but also the haematopoietic stem and progenitor cell niche within the bone marrow, haematological toxicity is the most commonly encountered side effect, which often results in fatal neutropenic sepsis [124]. The resultant cytopenia requires that the treatment has to be followed by autologous or allogenic stem cell transplant [26].

Hermann et al. treated three patients with advanced multiple myeloma and there was a good response initial response in two patients with improvement in the ^18^F-FDG positive lesions [125]. Two of the three patients died, one as a result of disease relapse and the other due to neutropenic sepsis. Maurer et al. treated 22 patients with lymphoproliferative or myeloid malignancies and, even though all patients developed cytopenia, nephrotoxicity and hepatotoxicity were low (seen in 2/25 treatments) and only one patient developed grade three kidney failure due to tumour lysis syndrome [124]. Lapa et al. administered ^177^Lu- and ^90^Y-Pentixather to six heavily pretreated relapsed multiple myeloma and acute leukaemia patients and also reported that the treatment was tolerated with a partial response in 2/6 patients and mixed response in 2/6 patients. However, all patients developed aplasia and required stem cell transplantation [126]. Unfortunately, 3/6 patients succumbed to neutropenic sepsis and 3/6 died due to disease progression with a median progression-free survival of 62 d (range, 29–110 d) and median overall survival of 76 d (range, 29–334 d). However, these were all heavily pretreated patients with no other alternative treatment options. Thus far there have been no reports of targeted radionuclide therapy use in solid tumours, and it is likely that haematological toxicity will limit the application of CXCR4 radionuclide therapies to patients with no other treatment options and who will need an autologous or allogenic stem cell transplant [24].

## 4. Conclusions

CXCR4 expression forms an integral part of tumour survival and spread in several solid malignancies, and its expression is generally associated with more aggressive cancers, a poorer prognosis and high potential for metastases and/or recurrence.

The most promising applications in solid tumours appear to be theranostic approaches to the treatment of refractory advanced cholangiocarcinoma, adrenocortical carcinoma, vestibular schwannoma, small cell lung cancer, oesophageal cancer and high-grade neuroendocrine tumours, to select patients likely to benefit from therapies targeting CXCR4 including targeted radionuclide therapies with ^177^Lu- and ^90^Y-Pentixather in combination with stem cell transplantation in a selected group of patients with no other therapeutic options.

Imaging with a CXCR4-targeting tracer generally results in a lower SUVmax and lower target-to-background ratios when compared to ^18^F-FDG PET. However, in oesophageal and lung cancer, ^68^Ga-Pentixafor uptake has been demonstrated in ^18^F-FDG negative lesions, which requires further investigation.

Although ^68^Ga-Pentixafor-based imaging currently plays a complimentary role to ^18^F-FDG PET/CT in the staging of solid tumours, it may provide important prognostic information and aid in the selection of patients who may benefit from therapies targeting CXCR4 and in treatment response evaluation.

## Figures and Tables

**Table 1:** Potential applications of CXCR4 in solid tumours.

Tumour	CXCR4 Immunohistochemistry	Pentixafor	Pentixafor vs. FDG	Potential Application
HNSCC	+ in high grade tumours [34]	2 patients-low uptake [84]	No published data	Select potential candidates for anti-CXCR4 therapies
Glioblastoma	+++ in high grade tumours [37]	Low–moderate uptake [23]		Select potential candidates for anti-CXCR4 therapies
Oesophageal cancer	++ in 38–50% [41,43]	Most lesions + [45,46]	FDG > Pentixafor [46,47] (7/26 lesions Pentixafor +, FDG −) [46]	Select potential candidates for anti-CXCR4 therapies Monitor treatment response Alter treatment plan (upstaging) [45]
NET	Weak–moderate expression	80% of Gr3 NET +, 50% Gr 2, Gr 1 neg [98]	Inferior to FDG and SSTR [47]	Role in higher grade tumours Prognosis Select potential candidates for anti-CXCR4 therapies
Lung cancer	+ [50]	SCLCa higher than NSCLCa [51] Mesothelioma controversial [52,53]	More sensitive than FDG in 6/10 patients [49]	More sensitive than ^68^Ga DOTANOC Select potential candidates for anti-CXCR4 therapies Monitor treatment response
Osteosarcoma	+ 68% of 36 tumours Worse prognosis [55,56]	No published data	No published data	Prognosis [54] Potentially select patients for CXCR4-targeted therapy Monitor treatment response
Vulva cancer	+ correlates with poorer prognosis [59]	No published data	No published data	Differentiate tumour from premalignant lesion [59] Prognosis Select patients who may benefit from therapy
Cervix	+ = poorer prognosis [62]	No data	No data	Prognosis
Ovarian	+ primary = more advanced disease, poorer prognosis [63]	No data	No data	Prognosis
Breast	+ but no correlation with molecular subtype, Ki67 or ER/PR [70,71,73]	9/13 + [19] Recurrence metastasis 5/5 [27] 4/7 metastatic lesions	No data	Potentially select patients for therapy Monitor treatment response
Prostate cancer	+ = poorer prognosis	++ In tumour models [77] Bladder activity seen	No published data	Insufficient evidence
Vestibular schwannoma	+++ correlates with tumour invasiveness [80]	6/6 sufficient uptake TBR matched w IHC [78]	No published data	Potentially to select patients for therapy Prognosis
Adrenocortical cancer	+++ 9/18 samples + Ki67, poorer prognosis [82]	+ in 6 patients adrenocortical [86]	Pentixafor + in 2/9 FDG negative lesions [85] FDG superior to Pentixafor 9/22	Prognosis Treatment response Select patients for Rx [83]
Colorectal	+ in 76 samples = metastasis = aggressiveness [88,89]	Low to moderate 8 pts [90]	No published data	Potentially to select patients for therapy
Neuroblastoma	+ = tumour aggressiveness [13,94]	No published data	No published data	Potentially to select patients for therapy Prognosis
HCC	+ 65/78 patients [97]	Limited data + 2/5 pts [27,98]	No published data	Potential for selecting pts who may benefit from Rx
Melanoma	+ = higher tumour stage Mets > primary [99,101,102]	Limited data + 4/4 metastasis [27]	FDG SUVmax slightly higher [27]	Select potential candidates for anti-CXCR4 therapies
Cholangiocarcinoma	49% (122 pts) +++ = poorer prognosis [106,107]	3 cases = high uptake [98]	No published data	Prognosis Select potential candidates for anti-CXCR4 therapies
Pancreatic cancer	+ = poorer prognosis [109,110]	Low–moderate uptake 12 patients (TBR 2.92- >4) [84,98]	No published data	
Renal cell cancer	+++ with metastasis and poorer prognosis [113,114]	Low [98]	n/a	May be a marker of disease aggressiveness Select potential candidates for anti-CXCR4 therapies
Gastric cancer	+ = poorer prognosis [87,117,118]	No published data	No published data	Potentially select patients who may benefit from Rx

Key: − no uptake, + low uptake, ++ moderate, +++ high uptake, Rx: CXCR4-targeted therapies.

## Abbreviations

ACC	Adrenocortical carcinoma
BBB	Blood–brain barrier
CAFs	Cancer-associated fibroblasts
CXCR4	Chemokine receptor 4
DOTATOC	DOTA-D-Phe-Tyr3-octreotide
FDG	Fluorodeoxyglucose
GBM	Glioblastoma
HCC	Hepatocellular carcinoma
HNSCC	Head and neck squamous cell carcinoma
IHC	Immunohistochemistry
MIF	Macrophage inhibitory factor
NEC	Neuroendocrine carcinomas
NETs	Neuroendocrine tumours
NF 2	Neurofibromatosis type 2
PDAC	Pancreatic ductal adenocarcinoma
PET	Positron Emission Tomography
PSMA	Prostate specific membrane antigen
OC	Oesophageal cancer
RCC	Renal cell carcinoma
RT	Radiation therapy
SCLC	Small cell lung cancer
SDF-1	Stromal cell-derived factor-1
SSTR	Somatostatin receptor
TAMs	Tumour-associated macrophages
TBR	Tumour to background ratio
TKIs	Tyrosine kinase inhibitors
TME	Tumour microenvironment
TNF	Tumour necrosis factor
VEGF	Vascular endothelial growth factor
VSCC	Vulvar squamous cell carcinoma
VS	Vestibular schwannomas
